# Effect of body fat mass loss on prognosis of radical resection for pancreatic ductal adenocarcinoma based on bioelectrical impedance analysis

**DOI:** 10.1186/s12893-024-02315-4

**Published:** 2024-01-11

**Authors:** Yoshiyuki Shibata, Takeshi Sudo, Sho Tazuma, Haruki Sada, Naoki Tanimine, Norimitsu Shimada, Hirofumi Tazawa, Takahisa Suzuki, Takashi Onoe, Yosuke Shimizu, Hirotaka Tashiro, Atsushi Yamaguchi, Shinya Takahashi

**Affiliations:** 1 Department of Surgery, National Hospital Organization Kure Medical Center and Chugoku Cancer Center, 3-1 Aoyama, Kure, Hiroshima, 737-0023 Japan; 2 Department of Gastroenterology, National Hospital Organization Kure Medical Center and Chugoku Cancer Center, 3-1 Aoyama, Kure, Hiroshima, 737-0023 Japan; 3 Department of Surgery, Graduate School of Biochemical and Health Science, Hiroshima University, 1-2-3 Kasumi, Minami-ku, Hiroshima, 734-8551 Japan

**Keywords:** Bioelectrical impedance analysis, Body composition, BFM, Prognosis, Pancreatic ductal adenocarcinoma

## Abstract

**Background:**

Few reports have performed a prognostic analysis based on bioelectrical impedance analysis in patients with radical resection of pancreatic ductal adenocarcinoma (PDAC), and its usefulness in prognostic analysis remains unclear. This study aimed to evaluate body composition changes in patients undergoing radical resection for PDAC and analyze its impact on prognosis.

**Methods:**

The medical records of radical resection for patients with PDAC were retrospectively reviewed, and the parameters of body composition, including body weight, skeletal muscle mass, body fat mass (BFM), and extracellular water-total body water ratio, from preoperatively to 12 months postoperatively, for each surgical procedure were measured based on direct segmental multifrequency bioelectrical impedance analysis with an InBody 770 (InBody Inc., Tokyo, Japan) device. The clinicopathological and prognostic factors were analyzed.

**Results:**

Among 79 patients who underwent radical resection for PDAC, 36 (46%), 7 (8%), and 36 (46%) underwent pancreatoduodenectomy, total pancreatectomy, and distal pancreatectomy, respectively. The multivariate overall survival analysis demonstrated that BFM loss percentage at 1 month postoperatively ≧14% (*p* = 0.021), lymph node metastasis (*p* = 0.014), and non-adjuvant chemotherapy (*p* <  0.001) were independent poor prognostic factors. Multivariate analysis revealed that preoperative BFM < 12 kg and preoperative albumin < 3.5 g/dL were independently associated with BFM loss percentage at 1 month postoperatively ≧14% (*p* = 0.021 and *p* = 0.047, respectively).

**Conclusions:**

Loss of BFM in the early postoperative period may have a poor prognosis in radical resection of PDAC.

**Supplementary Information:**

The online version contains supplementary material available at 10.1186/s12893-024-02315-4.

## Background

Surgical resection is the only curative treatment option for pancreatic ductal adenocarcinoma (PDAC). Additionally, multidisciplinary treatment, including neoadjuvant and adjuvant therapies, has become the standard for PDAC in recent years [1]. However, even if curative resection is performed, the 5-year overall survival (OS) rate remains low at 20.7–44.1% [1–3]. Previous reports have investigated the impact of preoperative nutritional evaluation and body composition evaluation on the long-term prognosis of PDAC. Non-invasive nutritional status assessment includes nutritional risk screening such as nutritional risk screening 2002 (NRS2002) [4]. It also includes the assessment of anthropometric parameters such as body weight (BW) and height to calculate body mass index (BMI) and body composition parameters comprising skeletal muscle mass (SMM), skeletal muscle mass index (SMI), and body fat mass (BFM). Presence of inflammation reduces serum albumin concentrations, regardless of the underlying nutritional status. Therefore, blood biochemical findings such as the Glasgow Prognostic Score (GPS), modified Glasgow Prognostic Score (mGPS) [5, 6], and Prognostic Nutritional Index (PNI) [7, 8] are recognized to be associated with nutrition risk rather than malnutrition itself. The evaluation of the psoas major [9, 10] and general skeletal muscles [11] using abdominal computed tomography (CT) images have reported that patients with a poor preoperative nutritional status and sarcopenia may have poor long-term prognosis. In addition, poor preoperative nutritional status, sarcopenia, and visceral obesity may be associated with postoperative complications [12, 13].

Bioelectrical impedance analysis (BIA) is a non-invasive method widely used for evaluating body composition parameters, including SMM, SMI, BFM, extracellular water-total body water ratio (ECW/TBW), and phase angle (PhA). However, there is a scarcity of reports showing the effect of early postoperative body composition changes assessed by BIA on the long-term prognosis of radical resection for PDAC. Therefore, this study aimed to evaluate the body composition changes in the early postoperative period following radical resection for PDAC and analyze their impact on prognosis.

## Methods

### Study design

We retrospectively reviewed the medical records of patients who underwent radical resection for PDAC at the Department of Surgery of the National Hospital Organization Kure Medical Center in Hiroshima, Japan, between August 2016 and July 2022. This Institutional Review Board of National Hospital Organization Kure Medical Center (2023–17) approved this study, which was conducted following the principles of the Declaration of Helsinki. In addition, all patients provided written informed consent.

### Neoadjuvant therapy

In our institution, neoadjuvant therapy (NAT) has been administered to patients with resectable PDAC since 2019, as well as to those with borderline resectable (BR) or locally advanced (LA) PDAC with arterial contacts, such as the superior mesenteric, hepatic, or celiac arteries, since 2016. Previously, all patients with resectable pancreatic cancer had received surgery upfront. NAT regimens for patients with resectable PDAC included gemcitabine/S-1, while those for patients with BR/LA PDAC included gemcitabine/nab-paclitaxel/S-1, gemcitabine/nab-paclitaxel, and 5-fluorouracil/leucovorin/irinotecan/oxaliplatin. No patient received radiation therapy during the study period.

### Perioperative nutritional support

We administered oral supplementation containing arginine, ω-3 fatty acids, and RNA (oral IMPACT®; Nestle Japan Co., Ltd., Tokyo, Japan) to all patients with PDAC for 5 days before surgery [14–16]. Moreover, we performed pancreatic enzyme replacement therapy (PERT) before and after surgery. Regarding this therapy, the patients received the pancreatic digestive enzyme supplement LipaCreon® (150 mg, 12 times/day; VIATRIS, Tokyo, Japan), particularly those with decreased serum lipase, decreased serum pancreatic amylase, or main pancreatic duct obstruction before surgery from 2021, and after surgery from 2016 in our department [17–19].

### Data collection

Patient demographic and clinicopathological data for each surgical procedure included age, sex, initial carbohydrate antigen 19–9 (CA19–9) level, preoperative PNI, GPS, NRS2002 [4], and resectability status according to the National Comprehensive Cancer Network guideline version 22,021 [20], operating time, operative blood loss, pathological tumor size, lymph node metastasis, and adjuvant chemotherapy. Postoperative complications were defined as those with a Clavien–Dindo classification ≧Grade III [21]. The body composition parameters included the SMM, SMI, BFM, ECW/TBW, and whole body PhA, and were measured preoperatively and at 1, 6, and 12 months postoperatively based on direct segmental multifrequency BIA with an InBody 770 device (InBody Inc., Tokyo, Japan). We measured body composition using InBody at more than 2 hours after breakfast without intentional physical activity at the morning of the examination. Each patient had their usual breakfast without restrictions on breakfast. The patients stood barefoot on the scale and aligned their heels with round electrodes. Thereafter, they held the hand electrode and placed their thumb on the round electrode. With the arm straight and not contacting with the body, the impedance of five body parts (i.e., right arm, left arm, trunk, right leg, and left leg) was measured at six different frequencies (1 kHz, 5 kHz, 50 kHz, 250 kHz, 500 kHz, and 1000 kHz), while the reactance of the five body parts at three frequencies (5 kHz, 50 kHz, and 250 kHz) was also measured. The BIA has been assessed in normal populations, athletes, elderly, and hemodialysis patients, and closely correlates with the gold standard measurement by dual energy X-ray absorptiometry, underwater weight method, and air displacement plethysmography [22–26]. This tool is not based on statistical data of any specific population. Therefore, it is capable of accurately assessing patients with very different physical types, whether obese, elderly or athletic [27, 28].

### Adjuvant therapy and surveillance

Adjuvant chemotherapy was recommended to all patients and was administered to those who were tolerant and consenting after radical pancreatectomy. Regular surveillance was performed via blood testing, which included tumor markers and multidetector CT at intervals of 3–6 months. When two or more modalities, such as magnetic resonance imaging or positron emission tomography-CT, indicated a recurrence or recurrent lesion at two different time points, recurrence was confirmed and recorded. The survival time after surgery and cause of death were also recorded for the patients who died, and the OS time and recurrence status were recorded for those who survived.

### Statistical analysis

Data are expressed as medians and ranges or as absolute values and percentages. The chi-square or Fisher’s exact test was used for the categorical variables, while the Wilcoxon two-sample test was used to compare continuous variables in the univariate analysis. A paired t-test with Bonferroni correction was used for body composition changes. Survival curves were obtained using the Kaplan–Meier method, and the log-rank test was used to compare distributions. The proportional hazard regression model (Cox regression) was used to perform multivariate survival analyses. Hazard ratios (HRs) and 95% confidence intervals (CIs) were calculated, and cutoff values were defined by receiver operating characteristic (ROC) curve analysis for 3-year OS after surgery. Preoperative low SMI was defined as SMI < 7.0 kg/m^2^ in male and SMI < 5.7 kg/m^2^ in female patients [29]. The cutoff PNI value was set at 45 as previously described [30]. All tests were two-sided, with statistical significance set at *p* <  0.05. All statistical analyses were performed using JMP statistical software (version 16.0; SAS Institute, Cary, NC, USA).

## Results

### Patients

A total of 79 patients underwent radical resection for PDAC. The clinicopathological characteristics of the patients for each surgical procedure are presented in Table 1. Among 79 patients who underwent radical resection for PDAC, 36 (46%), 7 (8%), and 36 (46%) underwent pancreatoduodenectomy (PD), total pancreatectomy (TP), and distal pancreatectomy (DP), respectively. Preoperative BMI and preoperative serum albumin levels were significantly lower in the PD group among the three groups (21.2, 21.5, 23.1 kg/m^2^, respectively; *p* = 0.033; and 3.6, 3.9, and 3.8 g/mL, respectively; *p* = 0.045). The number of patients with preoperative NRS2002 ≧3 was significantly higher in the PD group compared to the other groups (*p* = 0.005). Operating time and operative blood loss were significantly higher in the TP group among the three groups (499, 515, and 373 min, respectively; *p* <  0.001; and 460, 640, and 200 mL, respectively; *p* <  0.001). The number of patients who underwent postoperative PERT was significantly lower in the DP group compared to the other groups (*p* <  0.001).

**Table 1:** Patients’ characteristics (*n* = 79)

	PD (*n* = 36)	TP (*n* = 7)	DP (*n* = 36)	*p*-value
Age, med (range), years	75 (42–84)	73 (68–79)	75 (52–89)	0.788
Sex, male, n (%)	19 (52%)	3 (43%)	19 (53%)	0.882
Neoadjuvant chemotherapy, n (%)	15 (42%)	4 (57%)	17 (47%)	0.727
Preoperative CA19–9 level, med (range), U/mL	23 (0–9784)	59 (3–727)	18 (0–1638)	0.454
Preoperative BW, med (range), kg	54 (37–85)	56 (47–73)	58 (45–70)	0.106
Preoperative BMI, med (range), kg/m^2^	21.2 (15.8–28.5)	21.5 (19.9–29.9)	23.1 (14.3–33.8)	0.033
Preoperative SMI, med (range), kg/m^2^	6.4 (4.5–8.6)	6.8 (5.6–7.1)	6.4 (5.1–9.0)	0.375
Preoperative serum albumin, med (range), g/ml	3.6 (2.2–4.3)	3.9 (2.5–4.3)	3.8 (2.5–4.8)	0.045
Preoperative PNI, med (range)	42.3 (26.1–52.0)	46.1 (29.2–54.6)	43.9 (34.7–66.3)	0.208
Preoperative GPS ≧1, n (%)	17 (47%)	2 (29%)	9 (25%)	0.131
Preoperative NRS2002 ≧3, n (%)	18 (50%)	1 (14%)	6 (17%)	0.005
Preoperative PERT, n (%)	3 (8%)	3 (43%)	3 (8.3%)	0.076
Resectability status, n (%)				
Resectable	30 (83%)	4 (57%)	28 (78%)	0.343
Borderline resectable / Unresectable	6 (17%)	3 (43%)	8 (22%)	
Operating time, med (range), min	499 (350–751)	515 (345–699)	373 (205–511)	< 0.001
Operative blood loss, med (range), ml	460 (50–2000)	640 (200–3570)	200 (30–1200)	< 0.001
Pathological tumor size, med (range), mm	27 (8–63)	40 (18–80)	26 (0–90)	0.165
Lymph node metastasis, n (%)	24 (67%)	4 (57%)	18 (50%)	0.355
Adjuvant chemotherapy, n (%)	34 (94%)	6 (87%)	29 (81%)	0.187
Postoperative PERT, n (%)	31 (86%)	7 (100%)	15 (42%)	< 0.001

*PD* pancreatoduodenectomy, *TP* total pancreatectomy, *DP* distal pancreatectomy, *med* median, *CA19–9* carbohydrate antigen 19–9, *U/mL* units/mL, *BW* body weight, *BMI* body mass index *SMI* skeletal muscle mass index, *PNI* Prognostic Nutritional Index, *GPS* Glasgow Prognostic Score, *NRS2002* Nutritional Risk Screening 2002, *PERT* pancreatic enzyme replacement therapy

### Body composition changes

**Fig. 1:**
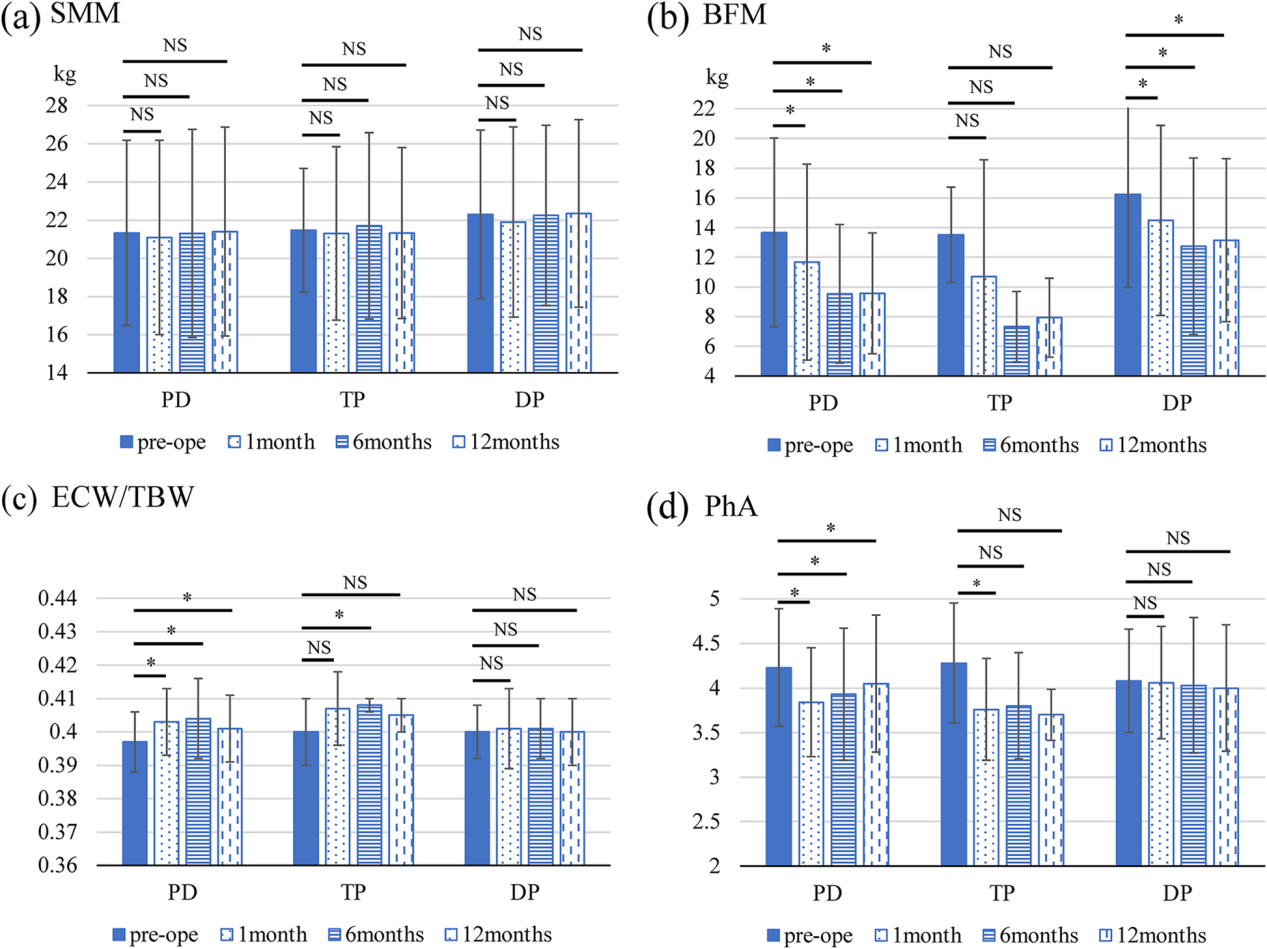
Mean body composition preoperatively and at 1, 6, and 12 months postoperatively for each surgical procedure (**a**) SMM, (**b**) BFM, (**c**) ECW/TBW, and (**d**) PhA. BFM, body fat mass; DP, distal pancreatectomy; ECW/TBW, extracellular water-total body water ratio; NS, not significant; PD, pancreatoduodenectomy; SMM, skeletal muscle mass; TP, total pancreatectomy; PhA, phase angle *, *p* < 0.05

The analysis of postoperative mean body composition showed that the mean SMMs in the PD group preoperatively and at 1, 6, and 12 months postoperatively were 21.3 ± 4.9, 21.1 ± 5.1, 21.3 ± 5.5, and 21.4 ± 5.5 kg, respectively; the corresponding values in the TP group were 21.5 ± 3.2, 21.3 ± 4.5, 21.7 ± 4.9, and 21.3 ± 4.5 kg, respectively; and, in the DP group, these values were 22.3 ± 4.4, 21.9 ± 5.0, 22.3 ± 4.7, and 22.4 ± 4.9 kg, respectively (Fig. 1a). No significant difference was observed for any period. The mean BFM in the PD group was 13.7 ± 6.4, 11.7 ± 6.6, 9.5 ± 4.7 kg, and 9.6 ± 4.1 kg; the corresponding values in the TP group were 13.5 ± 3.2, 10.7 ± 7.9, 7.3 ± 2.4, and 7.9 ± 2.7 kg; and, in the DP group, these values were 16.3 ± 6.3, 14.5 ± 6.4, 12.7 ± 5.9, and 13.2 ± 5.5 kg, respectively (Fig. 1b). The BFM in the PD and DP groups at 1, 6, and 12 months postoperatively significantly decreased compared to the preoperative BFM. The mean ECW/TBW in the PD group was 0.397 ± 0.009, 0.403 ± 0.010, 0.404 ± 0.012, and 0.401 ± 0.010; the corresponding values in the TP group were 0.400 ± 0.010, 0.407 ± 0.011, 0.408 ± 0.002, and 0.405 ± 0.005, respectively; and, in the DP group, these values were 0.400 ± 0.008, 0.401 ± 0.012, 0.401 ± 0.009, and 0.400 ± 0.010, respectively (Fig. 1c). The ECW/TBW in the PD group at 1, 6, and 12 months postoperatively and the corresponding values in the TP group at 6 months postoperatively significantly increased compared to the preoperative ECW/TBW. The mean PhA in the PD group preoperatively and at 1, 6, and 12 months postoperatively was 4.2 ± 0.7 and 3.8 ± 0.6, 3.9 ± 0.7, and 4.1 ± 0.8, respectively. The corresponding values in the TP group were 4.3 ± 0.7 and 3.8 ± 0.6, 3.8 ± 0.6, and 3.7 ± 0.3, respectively. Furthermore, in the DP group, these values were 4.1 ± 0.6 and 4.1 ± 0.6, 4.0 ± 0.8, and 4.0 ± 0.7, respectively (Fig. 1d). The PhA in the PD group at 1, 6, and 12 months postoperatively and the corresponding values in the TP group at 1 months postoperatively significantly decreased compared to the preoperative PhA value. The impedance and reactance of the five body parts are presented in Additional file 1.

### Survival analysis

**Fig. 2:**
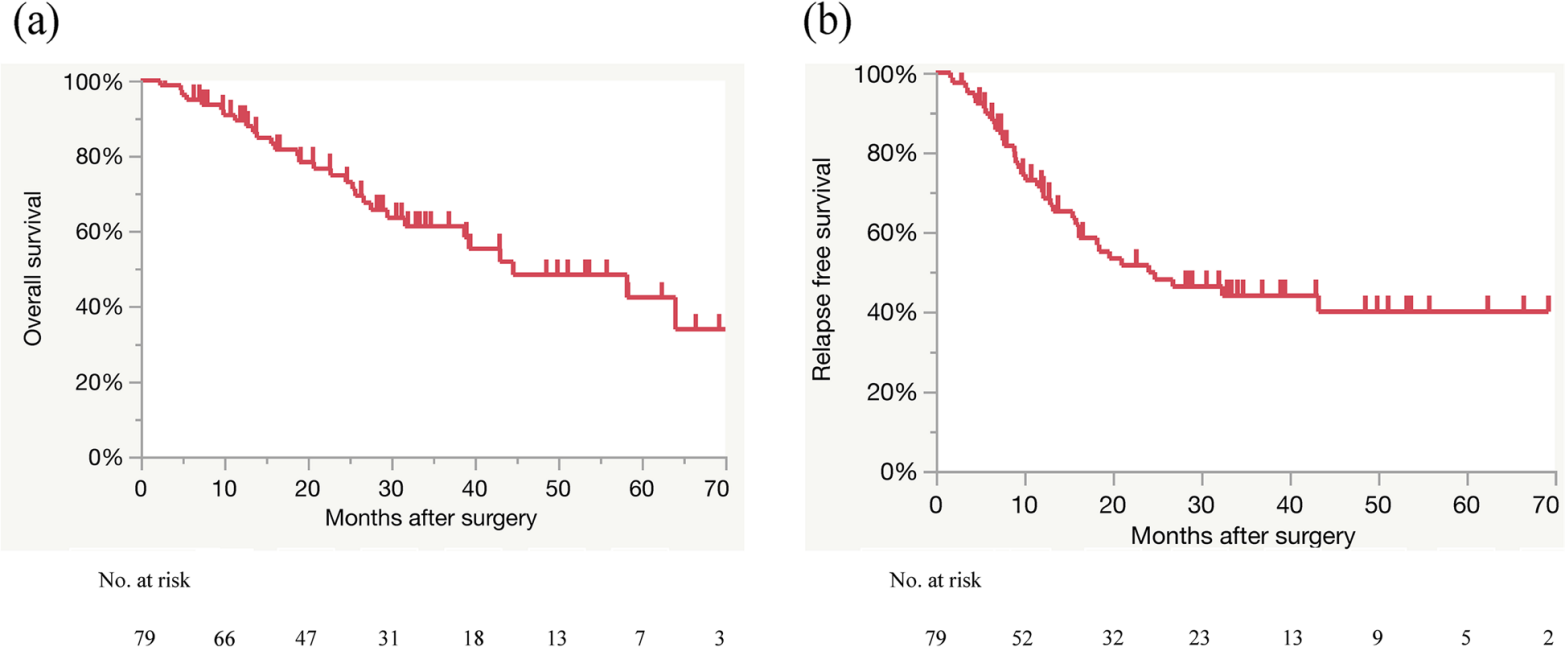
The survival curve for all patients (**a**) Overall survival after surgery (**b**) Relapse free survival after surgery

**Fig. 3:**
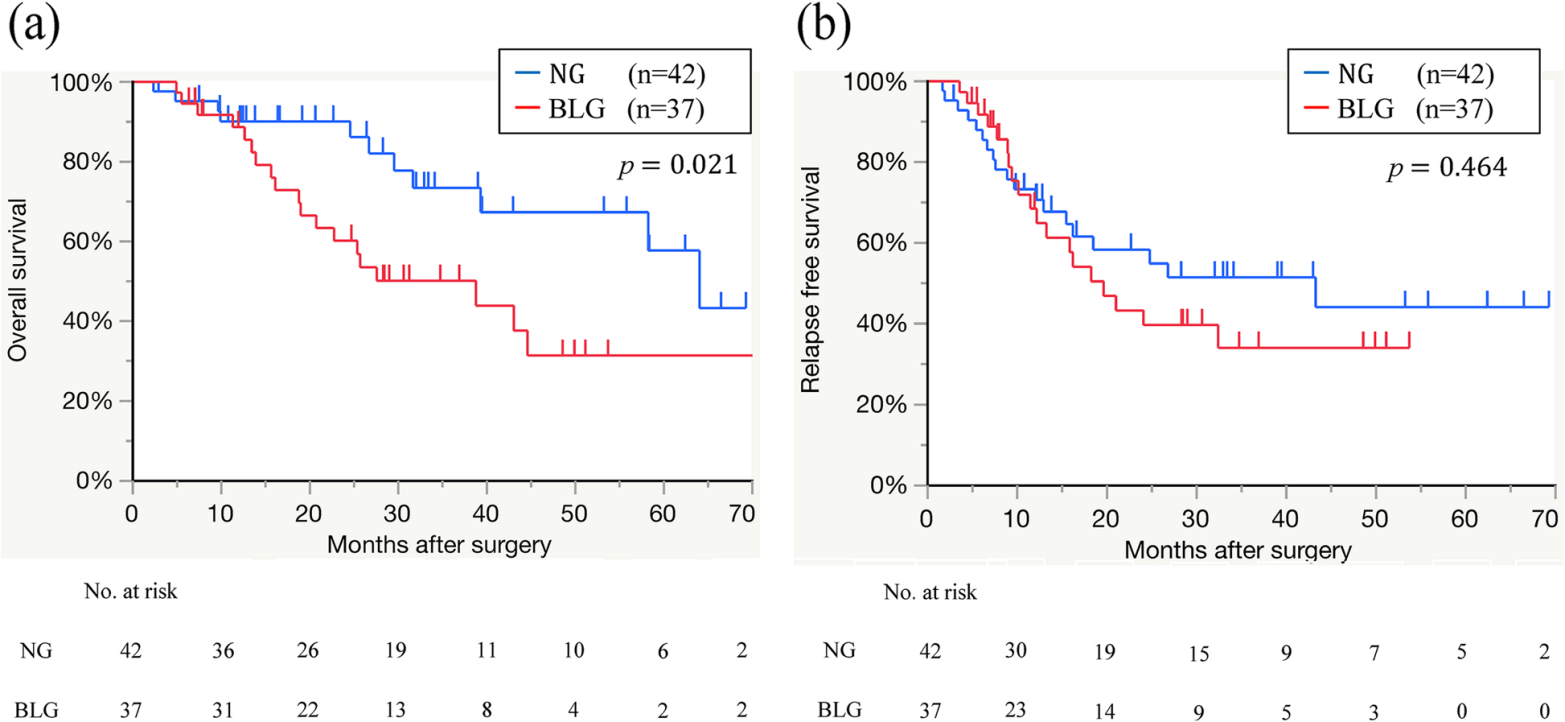
Survival curves stratified by BFM loss percentage ≧14% at 1 month postoperatively (**a**) The median overall survival rate after surgery was significantly worse in the BLG than in the NG (*p* = 0.021). (**b**) The relapse free survival after surgery stratified by the BLG or NG. There was no significant difference in median relapse free survival after surgery between the two groups (*p* = 0.464). BLG, body fat mass loss group; NG, normal group

The median follow-up period was 25.4 (range, 2.9–73.5) months, and the survival curve of all patients showed that the median survival time (MST) after surgery was 44.6 months (Fig. 2a). The postoperative 1-, 3- and 5-year survival rates were 89, 61, and 42%, respectively. The median relapse-free survival (RFS) after surgery was 24.1 months (Fig. 2b), and the postoperative 1-, 3- and 5-year RFS rates were 71, 44, and 40%, respectively. The univariate and multivariate OS analyses of the factors predictive of a poor prognosis are shown in Table 2. Univariate OS analysis of poor prognostic factors demonstrated that BFM loss percentage at 1 month postoperatively ≧14% (*p* = 0.021), lymph node metastasis (*p* = 0.030), and no-adjuvant chemotherapy (*p* <  0.001) were significantly associated with OS. Furthermore, multivariate analysis demonstrated that BFM loss percentage 1 month after surgery ≧14% (HR, 2.52; 95% CI, 1.15–5.55; *p* = 0.021), lymph node metastasis (HR, 3.61; 95% CI, 1.30–10.02; *p* = 0.014), and no-adjuvant chemotherapy (HR, 13.30; 95% CI, 4.08–43.41; *p* < 0.001) were independent poor prognostic factors. The survival curves of patients stratified by BFM loss percentage at 1 month postoperatively ≧14%, divided into the BFM loss group (BLG) or the normal group (NG), are shown in Fig. 3. The median OS rate after surgery was significantly worse in the BLG than in the NG (38.8 vs 64.0 months, *p* = 0.021) (Fig. 3a). The RFS postoperatively was stratified by the BLG or NG. There was no significant difference in the median RFS after surgery between the two groups (19.6 vs 43.3 months, *p* = 0.464) (Fig. 3b). The 86% of patients in the BLG and 88% of those in the NG underwent adjuvant chemotherapy (*p* = 0.830). The 51% of patients in the BLG and 45% of those in the NG recurred (*p* = 0.587). The multivariate analysis revealed that preoperative BFM < 12 kg and preoperative albumin < 3.5 g/dL were independently associated with BFM loss percentage at 1 month postoperatively ≧14% (odds ratio [OR], 4.03; 95% CI, 1.23–13.19; *p* = 0.021 and OR, 3.13; 95% CI, 1.02–9.63; *p* = 0.047, respectively) (Table 3).

**Table 2:** Univariate and multivariate overall survival analyses of poor prognostic factors (*n* = 79)

Factors	Univariate			Multivariate		
	Entire (*n* = 71)	MST (months)	*p*-value	HR	95% CI	*p*-value
Age, years						
< 80	59 (75%)	43.0	0.841			
≥ 80	20 (25%)	58.2				
Sex						
Male	41 (52%)	43.0	0.506			
Female	38 (48%)	58.2				
Neoadjuvant chemotherapy						
Yes	36 (46%)	64.0	0.281			
No	43 (54%)	39.3				
Preoperative CA19–9 level, U/mL						
< 100	54 (68%)	58.2	0.610			
≧100	25 (32%)	39.3				
Preoperative BMI, kg/m^2^						
< 18.5	7 (9%)	24.6	0.152			
≧18.5	72 (91%)	58.2				
Preoperative BMI, kg/m^2^						
< 25	64 (81%)	43.0	0.420			
≧25	15 (19%)	58.2				
Preoperative low SMI, kg/m^2^						
Yes	34 (43%)	44.6	0.495			
No	45 (57%)	58.2				
Preoperative BFM, kg						
< 12	20 (25%)	26.7	0.092			
≧12	59 (75%)	58.2				
Preoperative ECW/TBW						
< 0.4	46 (58%)	43.0	0.871			
≧0.4	33 (42%)	58.2				
BFM loss percentage 1 month after surgery, %						
< 14	42 (53%)	64.0	0.021	1.0		0.021
≧14	37 (47%)	38.8		2.52	1.15–5.55	
SMM loss percentage 1 month after surgery, %						
< 5	55 (70%)	44.6	0.785			
≧5	24 (30%)	64.0				
preoperative serum albumin, g/mL						
< 3.5	23 (%)	38.8	0.475			
≧3.5	56 (%)	58.2				
preoperative PNI						
< 45	44 (56%)	39.3	0.611			
≧45	35 (44%)	44.6				
Preoperative GPS						
0	51 (65%)	58.2	0.990			
≧1	28 (35%)	39.3				
Resectability status						
Resectable	62 (78%)	43.0	0.985			
Borderline resectable / Unresectable	17 (22%)	64.0				
Surgical procedures						
PD / TP	43 (54%)	44.6	0.852			
DP	36 (46%)	58.2				
Pathological tumor size, mm						
< 30	48 (61%)	58.2	0.053	1.0		0.253
≧30	31 (39%)	25.7		1.68	0.69–4.11	
Lymph node metastasis						
Positive	46 (58%)	39.3	0.030	3.61	1.30–10.02	0.014
Negative	33 (42%)	58.2		1.0		
Surgical margin						
Positive	16 (20%)	58.2	0.763			
Negative	63 (80%)	44.6				
Postoperative complication						
Yes	6 (8%)	–	0.221			
No	73 (92%)	43.0				
Adjuvant chemotherapy						
Yes	69 (87%)	64.0	< 0.001	1.0		< 0.001
No	10 (13%)	27.6		13.30	4.08–43.41	

*BFM* body fat mass, *BMI* body mass index, *CA19–9* carbohydrate antigen 19–9, *CI* confidence interval, *DP* distal pancreatectomy, *ECW/TBW* extracellular water-total body water ratio, *GPS* Glasgow Prognostic Score, *HR* hazard ratio, *MST* median survival time, *PD* pancreatoduodenectomy, *PNI* Prognostic Nutritional Index, *SMI* skeletal muscle mass index, *SMM* skeletal muscle mass, *TP* total pancreatectomy, *U/mL* units/mL

**Table 3:** Multivariate analyses of factors associated with BFM loss percentage 1 month after surgery ≧ 14%

Factors	Odds ratio (95% CI)	*p* value
Initial CA19–9 ≧37 U/ml	0.47 (0.17–1.32)	0.151
Preoperative BFM < 12 kg	4.03 (1.23–13.19)	0.021
Preoperative serum albumin < 3.5 g/dl	3.13 (1.02–9.63)	0.047
Surgical margin positive	0.36 (0.10–1.33)	0.126
Postoperative complication	0.38 (0.05–2.93)	0.350
Serum CRP at 1 month after surgery ≧1 mg/dl	2.07 (0.56–7.62)	0.275

*CI* confidence interval, *BFM* body fat mass, *CA19–9* carbohydrate antigen 19–9, *U/mL* units/mL, *CRP* C-reactive protein

## Discussion

This study revealed that BFM loss in the early postoperative period in patients having undergone radical resection for PDAC may lead to poor prognosis based on direct segmental multifrequency BIA assessment. Currently, there are no established methods for evaluating the nutritional status and body composition in patients with PDAC; however, there have been various reports about the impact on long-term prognosis after surgery for PDAC based on blood biochemistry findings or body composition changes. Previously, Kim et al. reported that a low PNI change (− 1.94) between pre- and post-neoadjuvant chemotherapy (NAC) was an independent risk factor for the OS of patients with resected PDAC following NAC. In the preoperative setting, improving the PNI can improve the long-term oncologic outcomes of PDAC [7]. Abe et al. [30] found that preoperative PNI ≧45 in patients undergoing surgical resection for PDAC may be associated with good prognosis. In addition, Yamada, et al. indicated that a GPS score of 2 points was a significant independent poor prognostic factor in patients that underwent pancreatic cancer resection [31]. Regarding body composition changes, many studies have used abdominal CT imaging. In previous reports, preoperative visceral adiposity and sarcopenic visceral obesity, as well as low muscle mass and quality, were found to be associated with mortality and recurrence after resection of PDAC [11], and postoperative skeletal muscle changes were independently associated with prognostic factors [32].

BIA is widely used as it is a simple and non-invasive method that provides accurate results [33, 34]. Several reports have evaluated postoperative changes in body composition using BIA and analyzed the risk factors for postoperative complications in patients with gastric cancer [35–37]. Moreover, in patients with colorectal cancer, a preoperative low SMI based on the BIA method was considered a risk factor for postoperative ileus [38], and preoperative visceral fat area measured by BIA was an independent prognostic factor for disease-free survival [39]. Only a few studies have investigated the relationship between the prognosis after radical resection of PDAC and body composition changes assessed by BIA. Gupta et al. reported that the phase angle used as an index for evaluating cell membranes and intra- and extracellular water balance was a strong prognostic indicator in advanced PDAC, and patients with a phase angle > 5.0° had a significantly better prognosis than those with a phase angle < 5.0° (MST, 10.2 vs 6.3 months, *p* = 0.02) [40]. Additionally, Tozuka, et al. revealed that SMI obtained by BIA may be a predictor of treatment response and prognosis in patients with advanced PDAC who underwent gemcitabine plus nab-paclitaxel combination therapy, and the progression-free survival in the sarcopenia group was significantly lower compared with that of the non-sarcopenia group (4.4 vs 6.4 months, *p* = 0.03) [41]. In the current study, the SMM did not change significantly, while the BFM changed significantly from preoperatively to 12 months postoperatively; therefore, we speculated that the rate of BFM change may be a sensitive reflection of malnutrition related to prognosis. Among the 79 patients who underwent radical resection for PDAC, 53 patients (67%) (31, 7, and 15 patients in the PD, TP, and DP groups, respectively) underwent postoperative PERT. Moreover, in our department, from the day after surgery, the patients could walk around the ward with a physical therapist as postoperative physical exercise. This may have led to the maintenance of SMM. Although there was no statistically significant difference, serum C-reactive protein 1 month after surgery measuring ≧1 mg/dl tended to be involved in BFM loss percentage 1 month after surgery ≧ 14%. Prolonged inflammation after surgery may have contributed to BFM loss. Recent reports have stated that marked loss of muscle, visceral fat, or subcutaneous fat significantly predicted shorter disease-free survival (DFS) and OS among patients with gastric cancer who underwent gastrectomy [42], and the marked loss of adipose tissue was found to be associated with a poor nutritional status. Poorer OS and DFS rates were observed in patients with marked visceral adipose tissue loss and subcutaneous adipose tissue loss during neoadjuvant treatment [43]. However, to our knowledge, no report to date has shown the relationship between postoperative BFM loss and prognosis of radical resection for PDAC. Therefore, BFM loss in the early postoperative period may reflect poor general condition.

Among gastrointestinal cancer procedures, pancreatectomy for PDAC is a highly invasive surgery. Body composition changes result from various mechanisms, such as hyper-catabolism associated with the inflammatory reactions caused by surgical stress, reduced food intake, and a decline in activity. In cases of surgical stress, immune cells produce cytokines that act as mediators of both immune and systemic responses to injury. Muscle catabolism is accelerated by the cytokines produced during and after surgery, leading to a decrease in muscle content after surgery [44]. Cytokines associated with inflammatory reactions could possibly cause the adverse feedback action of leptin on the hypothalamus, causing a loss of appetite [45]. Furthermore, patients who underwent PDAC lose the largest percentage of BW until discharge from the department compared to those with gastric cancer and colorectal cancer [46]; however, according to another previous report considering operative methods, BW change 12 months after surgery in the PD group was significantly lower than that found in the total gastrectomy and distal gastrectomy groups [47]. Therefore, there may be differences in the way body composition changes based on the postoperative period (until discharge from the department or 12 months after surgery) and operation methods such as PD, total gastrectomy, or distal gastrectomy. In our study, patients who underwent TP tended to lose more weight postoperatively than those who underwent PD or DP, without a significant difference, because of the small number of patients. This probably reflects the fact that most of the cases in the TP group included patients with highly malignant tumors with combined resection of the portal vein, and the surgical invasion was larger than that in corresponding patients who underwent PD or DP. There is no established method to reduce postoperative BW and BFM loss; in fact, our study showed that preoperative BFM < 12 kg and preoperative albumin < 3.5 g/dL were independently associated with BFM loss percentage at 1 month postoperatively ≧14%. However, no association with postoperative complications was found. This suggests the importance of preoperative factors and maintaining performance status and exercise tolerance. The maintenance of body composition changes in SMM and BFM through nutritional support comprised preoperative immunonutrition [16], PERT [17, 18], exercise therapy, and rehabilitation before and after surgery, which may lead to reducing postoperative complications and improving prognosis. Ausania et al. reported that prehabilitation that includes preoperative exercise therapy for patients with pancreatic or periampullary tumors who were candidates for pancreaticoduodenectomy was significantly associated with lower delayed gastric emptying incidence [48]. Additionally, Bundred et al. [49] indicated that prehabilitation programs may improve postoperative outcomes following pancreatic surgery. Current prehabilitaton programs for patients undergoing pancreatic resection include diverse exercise regimens, and there is no consensus regarding timing or length of prehabilitation; thus, there is a need to establish standardized prehabilitation programs in pancreatic surgery. Moreover, the importance of enhanced recovery after surgery protocols [50] for patients with PDAC has been emphasized.

This study had some limitations. First, it only included patients from a single institution, and the number of patients who underwent radical resection for PDAC was small. Hence, further studies using multicenter data or a nationwide database with a larger number of patients are needed. Second, this study had a retrospective design based on data not primarily meant for research. Third, we determined cutoff values based on the ROC curves for 3-year OS after surgery; however, no specific cutoff values for the PDAC population exist and, therefore, further investigations are necessary.

## Conclusion

BFM loss in the early postoperative period in patients having undergone radical resection for PDAC may lead to poor prognosis based on direct segmental multifrequency BIA assessment.

### Supplementary Information

**Additional file 1.**

## Data Availability

The datasets used and/or analyzed during the current study are available from the corresponding author on reasonable request.

## Abbreviations

BFM: Body fat mass
BIA: Bioelectrical impedance analysis
BLG: Body fat mass loss group
BMI: Body mass index
BR: Borderline resectable
BW: Body weight
CA19–9: Carbohydrate antigen 19–9
CI: Confidence intervals
CT: Computed tomography
DP: Distal pancreatectomy
ECW/TBW: Extracellular water-total body water ratio
GPS: Glasgow Prognostic Score
HR: Hazard ratios
LA: Locally advanced
mGPS: modified Glasgow prognostic score
MST: Median survival time
NAC: Neoadjuvant chemotherapy
NAT: Neoadjuvant therapy
NRS2002: Nutritional risk screening 2002
NG: Normal group
OS: Overall survival
PD: Pancreatoduodenectomy
PDAC: Pancreatic ductal adenocarcinoma
PERT: Pancreatic enzyme replacement therapy
PhA: Phase angle
PNI: Prognostic nutritional index
RFS: Relapse-free survival
ROC: Receiver operating characteristic
SMI: Skeletal muscle mass index
SMM: Skeletal muscle mass
TP: Total pancreatectomy
