# Supplementary material for: Effect of body fat mass loss on prognosis of radical resection for pancreatic ductal adenocarcinoma based on bioelectrical impedance analysis
Source: BMC Surg. 2024 Jan 11;24:19. doi: 10.1186/s12893-024-02315-4 (PMC10785500; doi:10.1186/s12893-024-02315-4)
Supplement: Supplementary file 1 — Additional file 1. [file 12893_2024_2315_MOESM1_ESM.docx]

**The impedance and the reactance of the five body parts (n=79)**

|  | **Pre-ope** | **1 months** | **6 months** | **12 months** |
| --- | --- | --- | --- | --- |
| 1kHz-RA Impedance, med (range) | 357.8 (246.0-515.9) | 386.5 (230.2-553.9) | 353.8 (213.3-578.7) | 346.3 (264.8-523.1) |
| 1kHz-LA Impedance | 354.7 (239.1-506.3) | 382.5 (255.6-535.4) | 343.6 (205.0-561.7) | 358.9 (254.4-495.8) |
| 1kHz-TR Impedance | 24.8 (14.7-355.7) | 21.8 (11.1-39.3) | 22.8 (9.0-348.8) | 22.8 (13.0-32.1) |
| 1kHz-RL Impedance | 250.1 (141.9-377.5) | 271.5 (131.2-467.1) | 236.5 (139.0-357.2) | 242.4 (132.6-319.8) |
| 1kHz-LL Impedance | 250.6 (22.1-362.2) | 274.1 (163.2-352.8) | 239.4 (20.1-348.9) | 247.8 (126.2-312.7) |
| 5kHz-RA Impedance | 351.8 (233.6-506.0) | 378.4 (226.3-546.1) | 349.9 (209.2-568.7) | 341.6 (262.8-514.0) |
| 5kHz-LA Impedance | 349.5 (225.3-496.0) | 375.6 (251.2-527.5) | 338.8 (200.7-549.3) | 350.6 (252.6-485.0) |
| 5kHz-TR Impedance | 24.2 (14.4-323.9) | 21.3 (11.0-31.5) | 22.3 (9.0-339.2) | 22.4 (12.9-32.1) |
| 5kHz-RL Impedance | 246.4 (139.7-369.5) | 267.3 (129.6-354.9) | 232.8 (137.6-348.6) | 239.7 (132.0-314.7) |
| 5kHz-LL Impedance | 247.2 (19.5-355.2) | 269.8 (161.5-348.3) | 236.1 (17.4-343.3) | 244.1 (125.6-307.4) |
| 50kHz-RA Impedance | 324.0 (220.9-460.5) | 348.4 (211.8-504.2) | 324.3 (208.5-514.9) | 318.0 (243.8-467.6) |
| 50kHz-LA Impedance | 320.7 (211.7-448.8) | 346.3 (232.9-482.1) | 318.5 (199.7-492.8) | 327.8 (240.4-437.0) |
| 50kHz-TR Impedance | 21.5 (13.2-310.5) | 19.3 (10.2-28.6) | 20.0 (8.5-29.7) | 19.8 (12.1-29.5) |
| 50kHz-RL Impedance | 231.5 (133.2-327.5) | 250.4 (125.5-330.5) | 219.2 (13.7-309.1) | 224.9 (127.9-288.9) |
| 50kHz-LL Impedance | 229.2 (17.9-315.8) | 254.3 (156.1-326.9) | 220.4 (1.6-317.0) | 224.5 (121.7-282.7) |
| 250kHz-RA Impedance | 295.6 (217.4-421.0) | 324.8 (195.4-466.0) | 299.4 (7.8-471.0) | 292.3 (223.6-428.2) |
| 250kHz-LA Impedance | 294.3 (208.0-409.4) | 320.7 (214.9-442.7) | 291.9 (8.0-449.9) | 301.4 (222.3-402.9) |
| 250kHz-TR Impedance | 18.7 (11.6-297.3) | 16.3 (8.9-25.2) | 17.1 (7.8-31.0) | 17.0 (11.0-25.8) |
| 250kHz-RL Impedance | 214.8 (125.7-315.0) | 236.3 (119.9-308.1) | 204.5 (28.6-286.0) | 210.5 (122.1-265.9) |
| 250kHz-LL Impedance | 212.9 (15.7-298.1) | 236.8 (149.0-306.2) | 205.9 (3.3-293.0) | 207.6 (116.7-262.0) |
| 500kHz-RA Impedance | 286.1 (213.9-405.0) | 315.5 (189.6-450.4) | 289.4 (17.3-454.2) | 281.4 (216.0-412.6) |
| 500kHz-LA Impedance | 283.8 (202.1-394.0) | 309.3 (208.0-427.1) | 281.1 (17.9-433.4) | 290.2 (213.6-388.5) |
| 500kHz-TR Impedance | 17.0 (10.7-24.0) | 14.5 (8.0-22.8) | 15.2 (7.6-24.6) | 15.5 810.1-22.6) |
| 500kHz-RL Impedance | 210.5 (11.0-290.3) | 231.3 (118.9-302.6) | 200.3 (24.4-279.7) | 205.8 (120.2-261.3) |
| 500kHz-LL Impedance | 208.3 (0.0-292.7) | 232.3 (147.7-300.9) | 202.1 (4.3-286.5) | 203.8 (115.6-257.0) |
| 1000kHz-RA Impedance | 278.4 (5.6-388.3) | 307.2 (186.7-434.1) | 280.7 (16.4-439.2) | 270.1 (209.6-398.2) |
| 1000kHz-LA Impedance | 275.1 (6.3-378.3) | 301.0(203.8-413.7) | 274.9 (17.2-419.1) | 280.1 (206.4-373.7) |
| 1000kHz-TR Impedance | 14.9 (8.3-27.3) | 12.5 (5.5-19.0) | 13.0 (4.5-22.0) | 13.6 (7.1-18.2) |
| 1000kHz-RL Impedance | 206.4 (22.4-284.9) | 229.2 (115.3-299.6) | 198.2 (4.1-275.0) | 202.4 (118.0-258.2) |
| 1000kHz-LL Impedance | 207.2 (2.3-287.5) | 229.1 (144.0-296.8) | 198.5 (7.5-281.3) | 201.4 (113.2-255.4) |
| 5kHz-RA Reactance | 11.8 (6.7-20.8) | 12.5 (5.4-22.3) | 11.0 (3.8-20.8) | 11.1 (4.5-60.9) |
| 5kHz-LA Reactance | 11.1 (6.6-19.3) | 11.5 (6.5-24.7) | 10.5 (3.7-24.0) | 10.4 (3.5-20.8) |
| 5kHz-TR Reactance | 1.5 (0.0-26.0) | 1.2 (0.0-8.1) | 1.4 (0.0-4.7) | 1.4 (0.2-3.3) |
| 5kHz-RL Reactance | 6.7 (0.0-19.0) | 7.3 (0.0-13.6) | 6.1 (1.6-12.0) | 6.3 (2.1-13.7) |
| 5kHz-LL Reactance | 6.4 (2.9-16.7) | 6.9 (0.0-13.6) | 6.4 (1.5-11.6) | 6.4 (1.5-13.5) |
| 50kHz-RA Reactance | 24.1 (13.1-39.6) | 25.0 (13.7-37.6) | 22.0 (9.1-38.6) | 22.7 (11.0-38.0) |
| 50kHz-LA Reactance | 22.2 (13.7-38.0) | 23.6 (0.6-5.1) | 21.9 (8.9-39.5) | 22.2 (8.9-37.5) |
| 50kHz-TR Reactance | 2.6 (1.6-6.8) | 2.1 (5.3-25.5) | 2.4 (0.8-4.1) | 2.4 (0.8-3.7) |
| 50kHz-RL Reactance | 15.0 (3.5-33.5) | 15.8 (5.3-25.5) | 13.6 (4.4-25.2) | 14.2 (4.4-24.5) |
| 50kHz-LL Reactance | 15.2 (5.9-33.2) | 15.8 (6.0-24.8) | 14.2 (3.9-23.8) | 14.4 (4.2-24.2) |
| 250kHz-RA Reactance | 20.4 (3.1-30.4) | 20.9 (14.0-32.2) | 19.5 (10.7-31.2) | 19.9 (10.4-29.1) |
| 250kHz-LA Reactance | 20.5 (3.3-30.9) | 21.4 (14.0-34.0) | 19.8 (11.4-32.2) | 20.1 (9.7-30.0) |
| 250kHz-TR Reactance | 3.2 (1.4-13.2) | 3.1 (0.7-10.0) | 3.0 (0.6-6.1) | 3.0 (0.4-5.5) |
| 250kHz-RL Reactance | 14.6 (0.0-25.5) | 15.6 (6.1-21.9) | 13.7 (7.1-20.3) | 13.7 (5.5-20.1) |
| 250kHz-LL Reactance | 15.2 (7.7-24.8) | 15.7 (8.1-22.9) | 14.5 (6.3-20.9) | 14.9 (5.5-20.4) |

kHz, kilohertz; RA, right arm; LA, left arm; TR, trunk; RL, right leg; LL, left leg; Pre-ope, preoperative; 1 months, 1 months after surgery; 6 months, 6 months after surgery; 12 months, 12 months after surgery
